# P014: Acinetobacter is the most common pathogen associated with late-onset and recurrent ventilator-associated pneumonia in an adult ICU in Saudi Arabia

**DOI:** 10.1186/2047-2994-2-S1-P14

**Published:** 2013-06-20

**Authors:** HH Balkhy, A El-Saed, HM Al-Dorzi, R Khan, AH Rishu, YM Arabi

**Affiliations:** 1 Infection Prevention and Control Department, King Abdulaziz Medical City, Riyadh, Saudi Arabia; 2 Department of Infection Prevention and Control, King Abdulaziz Medical City, Riyadh, Saudi Arabia; 3 Intensive Care Department, King Abdulaziz Medical City, Riyadh, Saudi Arabia

## Introduction

The guidelines for initial empiric antimicrobial therapy for ventilator-associated pneumonia (VAP) are highly dependent on the type of causative pathogen and the time of diagnosis.

## Objectives

The objective was to examine the microbial causes of VAP and describe any variability by the timing of VAP onset and over-time.

## Methods

The current study was a prospective surveillance conducted at adult general ICU of a tertiary care hospital at Riyadh, Saudi Arabia. Microbial isolates obtained from blood and different respiratory specimens of patients diagnosed with VAP (using CDC definition) between August 2003 and June 2009 were included.

## Results

A total of 457 pathogens were identified during the study; 380 (83.2%) were associated with primary VAP and 77 (16.8%) were associated with recurrent VAP. Of primary VAP pathogens, 159 (41.8%) were associated with early-onset (<5 days) and 221 (58.2%) were associated with late-onset (≥5 days) VAP. The most common pathogens identified were *Acinetobacter*spp. (26.5%) followed by *Pseudomonas aeruginosa* (21.7%), *Staphylococcus aureus* including *MRSA* (15.3%), *Klebsiella* spp. (6.8%), *Haemophilus* spp. (6.1%), and *Enterobacter* spp. (5.0%). *Acinetobacter*spp. and *MRSA* were significantly associated with late-onset VAP while *Haemophilus* spp. and *Streptococcus pneumoniae* were significantly associated with early-onset VAP. *Acinetobacter* spp. was the only pathogen associated with recurrent VAP and its incidence showed a significant increasing trend during the study period. *Acinetobacter* spp. was significantly associated with prolonged ventilation, sedation, and nasogastric intubation.

## Conclusion

*Acinetobacter baumanii* is the most common and increasingly important pathogen associated with VAP in our patients, especially late-onset and recurrent VAP. Our ICU should continue actively screening for *Acinetobacter* in all admitted patients, shorten ventilation duration, minimize sedation, encourage oral gastric rather than nasogastric intubation, and improve currently implemented infection control measures including environmental disinfection.

## Disclosure of interest

None declared

